# PA-Win2: In Silico-Based Discovery of a Novel Peptide with Dual Antibacterial and Anti-Biofilm Activity

**DOI:** 10.3390/antibiotics13121113

**Published:** 2024-11-21

**Authors:** Jin Wook Oh, Min Kyoung Shin, Hye-Ran Park, Sejun Kim, Byungjo Lee, Jung Sun Yoo, Won-Jae Chi, Jung-Suk Sung

**Affiliations:** 1 Department of Life Science, Dongguk University-Seoul, Goyang 10326, Republic of Korea; oh5929@dongguk.edu (J.W.O.); shinmk94@dgu.ac.kr (M.K.S.); 2019111678@dongguk.edu (H.-R.P.); tpwns413@dongguk.edu (S.K.); 2 Research Institute, National Cancer Center, Goyang 10408, Republic of Korea; blee.inf@gmail.com; 3 Wildlife Quarantine Center, National Institute of Wildlife Disease Control and Prevention, Incheon 22382, Republic of Korea; lycosidae@korea.kr; 4 Species Diversity Research Division, National Institute of Biological Resources, Incheon 22689, Republic of Korea; wjchi76@korea.kr

**Keywords:** antimicrobial peptide, in silico analysis, deep learning prediction model, *Pseudomonas aeruginosa*, antibacterial activity, anti-biofilm activity, quorum sensing

## Abstract

**Background:** The emergence and prevalence of antibiotic-resistant bacteria (ARBs) have become a serious global threat, as the morbidity and mortality associated with ARB infections are continuously rising. The activation of quorum sensing (QS) genes can promote biofilm formation, which contributes to the acquisition of drug resistance and increases virulence. Therefore, there is an urgent need to develop new antimicrobial agents to control ARB and prevent further development. Antimicrobial peptides (AMPs) are naturally occurring defense molecules in organisms known to suppress pathogens through a broad range of antimicrobial mechanisms. **Methods:** In this study, we utilized a previously developed deep-learning model to identify AMP candidates from the venom gland transcriptome of the spider *Pardosa astrigera*, followed by experimental validation. **Results:** PA-Win2 was among the top-scoring predicted peptides and was selected based on physiochemical features. Subsequent experimental validation demonstrated that PA-Win2 inhibits the growth of *Bacillus subtilis*, *Escherichia coli*, *Staphylococcus aureus*, *Staphylococcus epidermidis*, *Pseudomonas aeruginosa*, and multidrug-resistant *P*. *aeruginosa* (MRPA) strain CCARM 2095. The peptide exhibited strong bactericidal activity against *P*. *aeruginosa*, and MRPA CCARM 2095 through the depolarization of bacterial cytoplasmic membranes and alteration of gene expression associated with bacterial survival. In addition, PA-Win2 effectively inhibited biofilm formation and degraded pre-formed biofilms of *P*. *aeruginosa*. The gene expression study showed that the peptide treatment led to the downregulation of QS genes in the Las, Pqs, and Rhl systems. **Conclusions:** These findings suggest PA-Win2 as a promising drug candidate against ARB and demonstrate the potential of in silico methods in discovering functional peptides from biological data.

## 1. Introduction

Over the past few decades, the misuse and overuse of antibiotics have contributed significantly to the emergence of antibiotic-resistant bacteria (ARB), which the World Health Organization (WHO) has identified as a critical threat to global health. The prevalence of ARB has limited treatment options for bacterial infections, leading to increased mortality rates and higher healthcare costs [1,2]. Thus, developing new therapeutic agents is crucial to overcome infections caused by ARB and to prevent further development of resistance to conventional drugs [3].

A biofilm is a complex community of bacteria enclosed in an extracellular matrix and poses a significant challenge in treating bacterial infections. Hence, since the biofilm increases resistance to antibiotics and host immune defenses, therapeutic strategies addressing both antibacterial activity and biofilm eradication are important. Quorum sensing (QS), a cell-to-cell communication system employed for biofilm development, regulates the expression of genes involved in biofilm matrix production, virulence factor synthesis, and drug resistance [4,5]. Thus, targeting the QS systems may be a potential approach to disrupting biofilm formation and enhancing the efficacy of antimicrobial agents.

Antimicrobial peptides (AMPs) are naturally occurring small peptides, generally ranging from five to 50 amino acids (AAs), that play a crucial role in the innate immune system of various organisms. AMPs are characterized by their cationic, amphiphilic properties, and α-helical structures, enabling them to serve as important defense molecules against bacteria, fungi, viruses, and other pathogens. Such physiochemical properties allow AMPs to selectively target the bacterial membrane, which is negatively charged due to the presence of anionic phospholipids, causing membrane disruption [6]. Additionally, AMPs can enter bacteria and interact with intracellular components such as DNA and proteins, inhibiting bacterial growth or survival [7]. Owing to their broad spectrum of antimicrobial mechanisms, AMP can be a promising solution for developing new therapeutics against ARB [8].

The discovery of novel AMPs can be facilitated by exploring natural sources such as animal venom. Animal venoms are rich in bioactive proteins, peptides, and small molecules, with peptides displaying multiple functionalities, including antimicrobial, anti-fungal, anti-inflammatory, and neuromodulatory activities [9,10,11]. As a result of their adaptation to a variety of environments and their long evolutionary history, spiders are known to possess a substantial diversity of peptide components [12,13]. For example, SNX-482 derived from the *Hysterocrates gigas* spider modulates the expression of immunoregulatory molecules in macrophages to have an anti-tumor profile, and HpTx1 derived from *Heteropoda venatoria* acts as an activator of Na_v_1.9 ion channels [14,15].

Traditional methods of identifying functional peptides from venom are time-consuming and laborious, requiring sufficient acquisition of venom samples followed by proteomic analysis. However, advancements and developments in artificial intelligence (AI) and in silico analyses have provided powerful alternatives [16]. In silico analyses allow for the evaluation of the structural and physiochemical properties of functional peptides, facilitating the discovery of potential candidates with desired characteristics [17,18,19]. In addition, machine learning (ML) and deep learning (DL) models can efficiently utilize large-scale biological datasets to predict potential AMPs [20,21].

In this study, a 20-mer peptide sequence was identified from the venom gland transcriptome of the spider *Pardosa astrigera* using a previously developed DL model [22,23]. Experimental validation revealed the peptide’s antibacterial activity against pathogenic strains, including multidrug-resistant *Pseudomonas aeruginosa* (MRPA). The peptide inhibited bacteria by disrupting the membrane and suppressing intracellular targets such as *polA*. Moreover, the peptide exhibited anti-biofilm activity by suppressing genes involved in QS in *P*. *aeruginosa* and MRPA. The study highlights the potential of in silico approaches for accelerating the discovery of new therapeutics to address the global threat of ARB.

## 2. Results

### 2.1. Identification of PA-Win2 from the Transcriptome of Pardosa Astrigera Venom Gland via In Silico Method

In order to discover a novel peptide with potent antibacterial activity, transcriptome data from the venom gland of *Pardosa astrigera* were analyzed [22]. A previously developed DL model that implements multi-task learning was used, predicting species-specific antimicrobial activity against *Bacillus subtilis*, *Escherichia coli*, *Pseudomonas aeruginosa*, *Staphylococcus aureus*, and *Staphylococcus epidermidis* [23]. Our objective was to identify peptide candidates exhibiting high overall prediction scores that may lead to the discovery of peptides with inhibitory effects against a broader range of bacterial strains. A sliding window of 20 AAs was applied to spider venom gland transcripts to broaden the range of prediction targets. Among the sequences analyzed, five with the highest overall prediction scores against all five strains were selected (Table 1).

**Figure 1:**
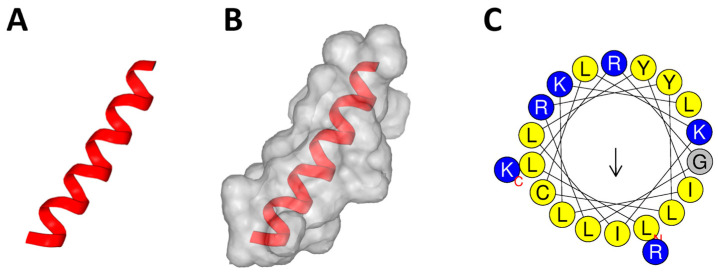
Structural analysis of PA-Win2. Structural modeling showed (**A**) the secondary structure and (**B**) the molecular surface of the peptide. (**C**) The amino acid configuration of PA-Win2 within the α-helical structure was presented.

Given that most AMPs exhibit a high net charge and good water solubility, these properties were calculated for the five peptide sequences. Although all peptide sequences showed a relatively high net charge of over +4.9, only a 20-mer sequence derived from the TBIU038647 transcript, which showed homology with peroxisomal membrane protein PEX13 of spider *Stegodyphus mimosarum*, was calculated to have good water solubility. The sequence was named PA-Win2 and its structural features were analyzed. Structural analysis revealed that PA-Win2 has an α-helical structure with a hydrophobic face consisting of residues through ILLC (Figure 1). Based on these in silico analyses, PA-Win2 was predicted to possess antibacterial activity with general characteristics of AMPs.

### 2.2. Evaluation of Antibacterial Activity and Cytocompatibility of PA-Win2

To verify the antibacterial activity of the peptide as predicted, minimum inhibitory concentration (MIC) and minimum bactericidal concentration (MBC) were determined against the abovementioned five bacterial strains. *B*. *subtilis* ATCC 6051, *E*. *coli* KCCM 11234, *P*. *aeruginosa* ATCC 9027, *S*. *aureus* KCCM 11335, and *S*. *epidermidis* ATCC 12228 were purchased from American Type Culture Collection (ATCC) or Korean Culture Center of Microorganisms (KCCM). Also, the clinical isolate MRPA CCARM 2095, which possesses resistance to ceftazidime, cefoperazone, ciprofloxacin, gentamicin, norfloxacin, and piperacillin, was obtained from the Culture Collection of Antimicrobial Resistant Microbes (CCARM) and tested. For comparison, conventional antibiotics such as ampicillin, streptomycin, tetracycline, and rifampicin were used. Prior to peptide treatment, the stability of PA-Win2 in tryptic soy broth (TSB) was tested, where no significant degradation of the peptide was observed during 24 h incubation (Figure S1).

MIC was assessed by incubating the bacteria with various concentrations (0.25–256 µg/mL) of PA-Win2 or antibiotics for 18 h. PA-Win2 showed lower MIC values than the common antibiotics in *B*. *subtilis*, *P*. *aeruginosa*, and MRPA CCARM 2095, while higher MIC values were observed for *S*. *aureus* and *S*. *epidermidis* (Table 2). Notably, PA-Win2 significantly inhibited MRPA CCARM 2095 with a MIC value of 2 µg/mL, exceeding all the tested antibiotics. In subsequent MBC tests, PA-Win2 exhibited similar or superior bactericidal properties against *E*. *coli*, *B*. *subtilis*, *P*. *aeruginosa*, and MRPA CCARM 2095 compared to the tested antibiotics. These results indicate that the antibacterial effects of PA-Win2 vary by strain, with efficient inhibition and bactericidal action at low concentrations against *B*. *subtilis*, *P*. *aeruginosa*, and MRPA CCARM 2095, but requiring higher concentrations against *S*. *aureus* and *S*. *epidermidis*.

**Figure 2:**
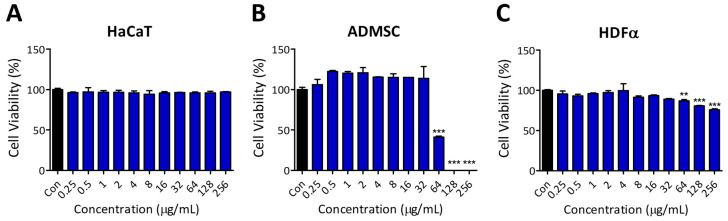
Evaluation of cytotoxicity in human normal cell lines upon PA-Win2 treatment. To assess the cytotoxicity of PA-Win2, various concentrations of the peptide were applied to human cell lines (**A**) HaCaT, (**B**) ADMSC, and (**C**) HDFα. While no significant cytotoxicity was observed in HaCaT cells, concentrations above 64 μg/mL significantly decreased cell viability in ADMSC and HDFα cells. ** *p* < 0.01 and *** *p* < 0.001 compared with control group. Con: control.

To evaluate the cytocompatibility of the peptide, we conducted cell viability assays using normal human cell lines. Cells were treated with the same peptide concentrations used in the antibacterial test (0.25 to 256 µg/mL) for 24 h. Epithelial keratinocytes HaCaT cells remained unaffected within the treated concentration range, whereas the viability of adipose-derived mesenchymal stem cells (hADMSCs) and adult dermal fibroblast (HDFα) cells decreased at concentrations above 64 µg/mL (Figure 2). Although PA-Win2 exhibited antibacterial activity against all tested strains, further experiments were conducted on *E*. *coli*, *B*. *subtilis*, *P*. *aeruginosa*, and MRPA CCARM 2095, for which both the MIC and MBC were determined to be below 32 ug/mL, without affecting human cells.

### 2.3. Bactericidal Activity of PA-Win2 by Membrane Depolarization

**Figure 3:**
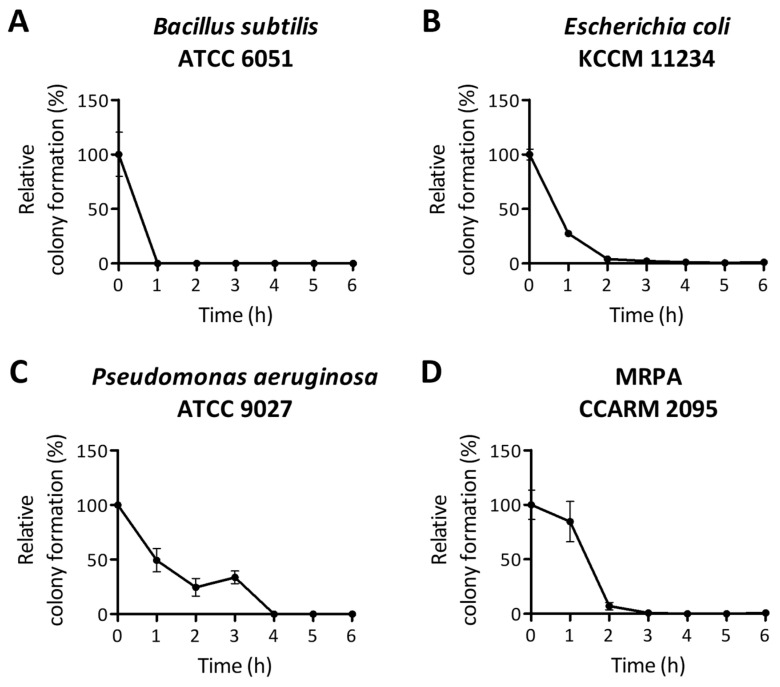
The bactericidal efficacy of PA-Win2 was evaluated using time-kill curve assays. The bactericidal effect of PA-Win2 was assessed against (**A**) *Bacillus subtilis*, (**B**) *Escherichia coli*, (**C**) *Pseudomonas aeruginosa*, and (**D**) MRPA CCARM 2095 using time-kill analysis. Bacterial strains were treated with 1× minimum bactericidal concentration for 6 h. Viable bacterial cells were measured every hour, where complete eradication was achieved across all strains within 4 h.

Time-kill assays were performed to evaluate the bactericidal effect of PA-Win2 over time using 1 × MBC values. For *B*. *subtilis*, complete killing was achieved within 1 h (Figure 3A). In the case of MRPA CCARM 2095, there was a significant reduction in colony formation at 2 h, with no colonies detectable after 3 h of peptide incubation (Figure 3D). Both *P*. *aeruginosa* and *E*. *coli* were completely eradicated after 4 h, where *E*. *coli* was observed for a drastic reduction of viable cells after PA-Win2 exposure for 1 h (Figure 3B,C).

**Figure 4:**
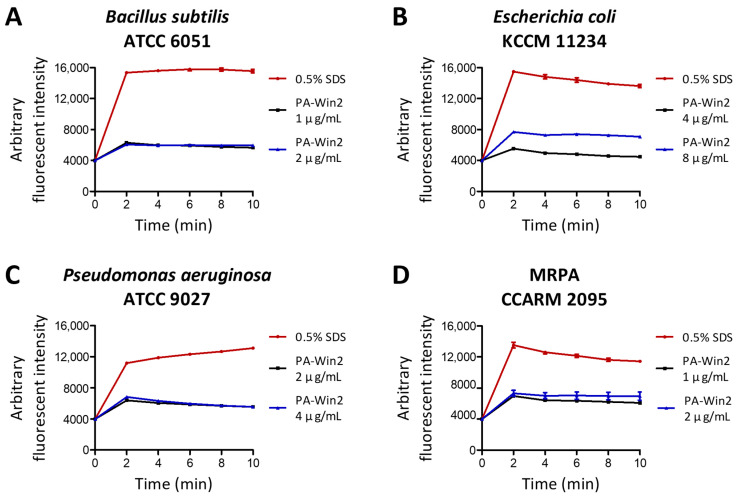
Bacterial cytoplasmic membrane disruption by PA-Win2 treatment. Membrane depolarization was assessed using 3,3′-dipropylthiadicarbocyanine iodide (DiSC_3_(5)) dye following treatment with PA-Win2. As a positive control, 0.5% sodium dodecyl sulfate (SDS) was used to cause complete bacterial membrane disruption. The effects of peptide or 0.5% SDS are presented as an arbitrary fluorescent intensity. PA-Win2 treatment induced a rapid release of DiSC_3_(5) in (**A**) *B*. *subtilis*, (**B**) *E*. *coli*, (**C**) *P*. *aeruginosa*, and (**D**) MRPA CCARM 2095.

Since the main mechanism of action of AMPs is to target the bacterial membrane, the effect of PA-Win2 on bacterial cytoplasmic membranes was evaluated using the fluorescent dye 3,3′-dipropylthiadicarbocyanine iodide (DiSC_3_(5)). The release of DiSC_3_(5) was measured after peptide treatment and presented along with 0.5% sodium dodecyl sulfate (SDS) control, which induced complete disruption of the bacterial membrane. Upon exposure to PA-Win2, all four bacterial strains exhibited rapid depolarization of their cytoplasmic membranes (Figure 4). DiSC_3_(5) release peaked within 2 min after peptide treatment of the peptide in all tested strains. PA-Win2 was demonstrated to effectively disrupt the bacterial membranes of pathogenic bacteria, contributing to rapid bactericidal activity.

### 2.4. Changes in P. aeruginosa and MRPA mRNA Expression upon PA-Win2 Treatment

Given the public health relevance of *P*. *aeruginosa* and MRPA, we further explored the antibacterial mechanisms of PA-Win2, which showed low MIC and MBC values and rapid bactericidal action. Reverse transcription-quantitative polymerase chain reaction (RT-qPCR) was conducted to assess expression changes in key genes associated with cell wall synthesis (penicillin-binding protein 2, *Pbp2*; UDP-N-acetylmuramoyl-L-alanine synthetase, *MurD*), DNA structure maintenance (DNA topoisomerase IV subunit A, *parC*; DNA gyrase subunit A, *gyrA*), DNA synthesis (DNA polymerase I, *polA*), RNA transcription (RNA polymerase β-subunit, *rpoB*), and protein synthesis (ribosomal protein S12, *rpsL*), following treatment with the peptide for 1 h.

**Figure 5:**
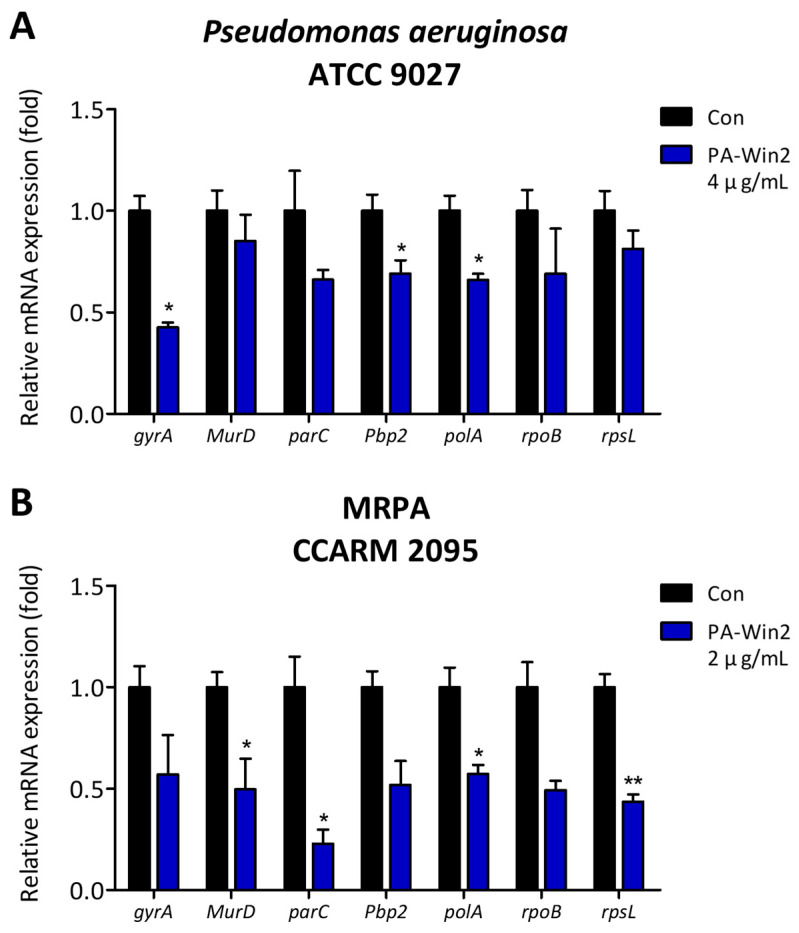
Suppression of gene expressions associated with bacterial growth and survival by PA-Win2. The mRNA expression levels of genes (*gyrA*, *MurD*, *parC*, *pbp2*, *rpoB*, *rpsL*) were analyzed using reverse transcription-quantitative polymerase chain reaction (RT-qPCR). mRNA expression in (**A**) *P*. *aeruginosa* and (**B**) MRPA CCARM 2095 was suppressed when treated with 4 μg/mL or 2 μg/mL PA-Win2, respectively. Significant differences are indicated as * *p* < 0.05 and ** *p* < 0.01 compared with the control group. Con: control.

In both bacterial strains, mRNA expression levels of all genes were reduced in the peptide-treated group compared with the control group. For *P*. *aeruginosa*, significant decreases were observed in *gyrA*, *pbp2*, and *polA* (Figure 5A). In MRPA CCARM 2095, genes *MurD*, *parC*, *polA*, and *rpsL* were downregulated by a minimum of approximately 2-fold (Figure 5B). Notably, *polA* expression was significantly suppressed in both strains, indicating that the peptide is involved in inhibiting DNA synthesis in *P*. *aeruginosa*.

### 2.5. PA-Win2 Inhibits Biofilm and QS Gene Expressions in P. aeruginosa and MRPA

**Figure 6:**
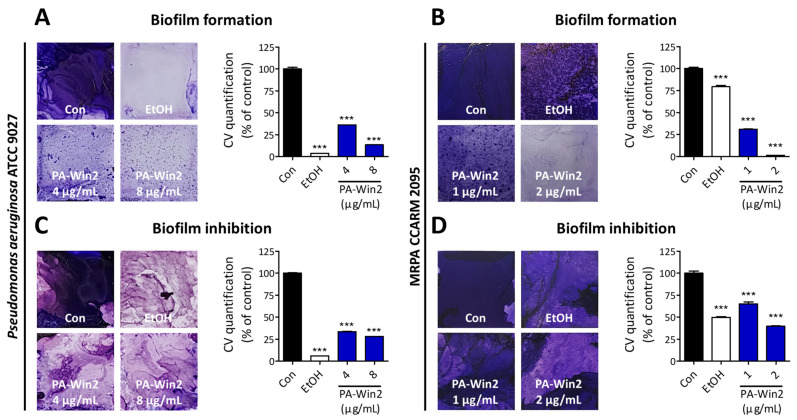
The effect of PA-Win2 on the formation and inhibition of biofilms. Representative images of crystal violet (CV) staining were used to assess biofilm formation and inhibition in (**A**,**C**) *P*. *aeruginosa* under static conditions and (**B**,**D**) MRPA CCARM 2095 under shaking conditions after treatment with PA-Win2. CV staining was eluted and quantified relative to the control group. The quantified data are presented in bar graphs. *** *p* < 0.001 compared with control group. Con: control, EtOH: Ethanol.

Biofilms are complex communities of microorganisms where bacteria attach to and grow on the surface, diminishing antibiotic efficacy and contributing to drug resistance [24]. To determine if PA-Win2 exerts anti-biofilm activity, biofilms were stained using crystal violet (CV). Biofilm formation and inhibition were tested using 70% ethanol and PA-Win2, which was applied either along with the biofilm formation or onto the already-formed biofilm. Under static conditions, the peptide inhibited biofilm formation and promoted biofilm degradation of *P*. *aeruginosa* at concentrations 4 µg/mL and 8 µg/mL (1× and 2 × MIC), while only minor suppression was observed in the case of MRPA CCARM 2095 at concentrations 2 µg/mL and 4 µg/mL (1× and 2 × MIC) (Figure 6A,C and Figure S2). Since MIC and MBC were determined under shaking conditions where contact between the peptide and bacterial cells is promoted compared with the static condition, we investigated the ability of *P*. *aeruginosa* and MRPA CCARM 2095 to form biofilms under shaking conditions. Unlike *P*. *aeruginosa*, MRPA CCARM 2095 formed biofilms during shaking with the same bacterial seeding density of static condition (Figure S3). The following PA-Win2 treatment confirmed significant inhibition as well as degradation of MRPA CCARM 2095 biofilm, exhibiting almost complete inhibition of biofilm formation at a concentration of 2 µg/mL (Figure 6B,D).

**Figure 7:**
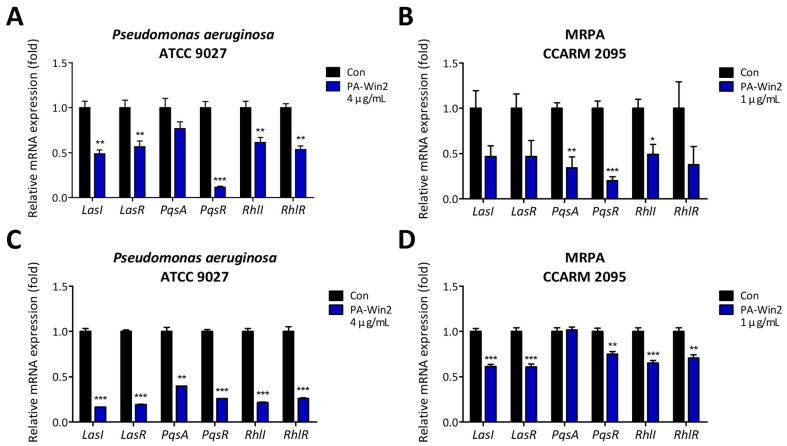
Downregulation of quorum sensing genes by PA-Win2 treatment. The mRNA expression levels of quorum sensing (QS) genes (*Lasl*, *LasR*, *PqsA*, *PqsR*, *RhlI*, *RhlR*) in *P*. *aeruginosa* and MRPA CCARM 2095 were evaluated following treatment with PA-Win2 at concentrations 4 μg/mL or 1 μg/mL, respectively. In *P*. *aeruginosa*, significant downregulation of QS genes was observed in both (**A**) biofilm formation and (**C**) biofilm inhibition, except for *PqsA* in the biofilm formation. In MRPA CCARM 2095, PA-Win2 induced suppression of QS-related gene in (**B**) biofilm formation and (**D**) biofilm inhibition, except for *PqsA*. Statistical significance is indicated as * *p* < 0.05, ** *p* < 0.01, and *** *p* < 0.001 compared with the control group. Con: control.

Further investigations into the expression of the QS genes were conducted using RT-qPCR after peptide treatment. Gene expression analysis indicated that treatment with 1 µg/mL PA-Win2 during biofilm formation significantly reduced QS gene expression in *P*. *aeruginosa* while having no significant reduction in MRPA CCARM 2095, consistent with the CV staining results (Figure 7A,B). In terms of biofilm inhibition, 1 µg/mL of PA-Win2 significantly reduced QS gene expression in both *P*. *aeruginosa* and MRPA CCARM 2095 compared with the control (Figure 7C,D). These findings not only supported the observed disruption and inhibition of biofilm by PA-Win2 but also suggested that the peptide may suppress bacterial virulence and resistance mechanisms through QS interference.

## 3. Discussion

The rise of ARB poses a significant threat to global health, demanding the urgent development of new antibiotics. As bacteria continue to evolve and acquire resistance to existing treatments, conventional antibiotics are becoming less effective against ARB infections, leading to increased morbidity, mortality, and healthcare costs [25,26,27]. The shortage of new therapeutic agents in development exacerbates the ARB crisis, emphasizing the need to identify new antimicrobials that are effective against resistant strains and possess mechanisms that reduce the potential for future resistance, thereby contributing to sustainable infection control [28]. To address this need, our study aimed to discover a novel AMP with potent antibacterial activity using animal transcriptome data and deep learning-based prediction alongside in silico analyses.

Recent advancements in computational methods have made in silico approaches valuable tools for identifying AMPs from biological data, including transcriptome. Traditional ML models like iAMPpred and AmPEP predict the functionality of peptides based on physicochemical characteristics and DL-based models such as AMP -BERT and Antimicrobial Peptide Scanner can process large-scale datasets to discover potential AMP sequences [29,30]. These technologies facilitate the detection of functional peptides from sequence information, significantly reducing the costs and time required for candidate selection and experimental validation. In this study, we employed a DL model developed from previous research to identify a new AMP sequence targeting *B*. *subtilis*, *E*. *coli*, *P*. *aeruginosa*, *S*. *aureus*, and *S*. *epidermidis* using multi-task learning [23]. The high overall prediction scores can be attributed to the relatively broad antibacterial activity, considering different approaches applied when dealing with each bacterial strain. We analyzed the venom gland transcriptome of the *Pardosa astrigera* spider and expanded the data by implementing a sliding window to truncate the transcripts by 20 AAs. While we utilized a 20-mer window in the study, applying varying lengths of truncation will yield a larger amount of data to be processed, facilitating research on functional peptide identification. Among the transcripts with top prediction scores, PA-Win2 was selected based on its high net charge and good water solubility, features typical of AMPs. Interestingly, the originating transcript of PA-Win2, TBIU038647, showed homology with *Stegodyphus mimosarum* spider peroxisomal membrane protein PEX13, which is not linked to antimicrobial activity. This finding implies that an in silico pipeline can enhance the utilization of biological data in the search for functional resources.

In the subsequent experimental validation, PA-Win2 exhibited antibacterial activity against all five strains mentioned above, as well as the MRPA strain CCARM 2095. For *B*. *subtilis*, *E*. *coli*, *P*. *aeruginosa*, and MRPA CCARM 2095, PA-Win2 demonstrated strong inhibition at low concentrations that were below the levels showing cytotoxicity to normal human cells. However, for *S*. *aureus* and *S*. *epidermidis*, MIC and MBC values were detected at higher concentrations that affected the viability of human cells. Since PA-Win2 showed stronger inhibitory action against Gram-negative strains, it was suggested that the peptide could potentially be developed as a strain-specific antimicrobial agent, especially for treating *P*. *aeruginosa* or MRPA infections. Furthermore, by employing sequence modifications based on functional predictions, it may be possible to design peptide derivatives with reduced cytotoxicity and enhanced antimicrobial efficacy. For example, increasing the number of positively charged residues through sequence substitution may enhance binding to bacterial membranes. This indicates that PA-Win2, discovered through an in silico process as an AMP, also opens up the possibility of generating additional peptides as an extension of this method.

After validating the antimicrobial activity of PA-Win2, we investigated its mechanism of action. The subsequent DiSC_3_(5) release assay revealed depolarization of the cytoplasmic membrane of pathogens following treatment with the peptide. This physical disruption of bacterial membranes leads to bacterial cell death and can prevent the accumulation of mutations and the development of antibiotic resistance. Additionally, resistance mechanisms involving enzyme production, enzymatic modification, and efflux pumps can be hindered, which increases antibiotic susceptibility [31,32,33]. For example, AMP P4-9 induced changes in the cell membranes of MRPA, enhancing the efficacy of the antibiotic novobiocin [34]. PA-Win2 showed the most significant membrane depolarization in MRPA CCARM 2095, suggesting its potential to effectively exert antimicrobial action and prevent further aggravation of drug resistance.

As AMPs are amphiphilic, they disrupt bacterial membranes and penetrate the cell, interacting with intracellular targets such as DNA and proteins, thereby affecting bacterial survival [7,35]. By examining the impact of PA-Win2 on these targets via RT-qPCR, we found that peptide treatment downregulated the genes associated with the cell wall organization, DNA structure and synthesis, RNA transcription, and protein synthesis in both *P*. *aeruginosa* and MRPA CCARM 2095. Notably, the expression of *polA*, crucial for DNA replication and repair, was significantly reduced by PA-Win2 in both strains [36]. This reduction suggests that DNA replication and subsequent biological activities are impeded by PA-Win2, making it difficult for bacteria to maintain genetic stability and adequately respond to external stresses [37,38]. The inability to repair damaged DNA could lead to abnormal functioning of overall gene regulation, possibly explaining the observed suppression of other survival-related genes. In summary, PA-Win2 demonstrated effective bactericidal activity, which was contributed to by both cell membrane disruption and intracellular targeting.

Biofilms form on surfaces by encasing bacteria within extracellular polymeric substances, protecting them from external stressors and reducing their susceptibility to antibiotics and immune cells [39,40]. Specifically, *P*. *aeruginosa* presents a substantial therapeutic challenge due to its ability to form biofilms that significantly enhance its antibiotic resistance, thereby making *P*. *aeruginosa* a leading cause of nosocomial infections and chronic infections [4]. In the biofilm formation and inhibition assays, PA-Win2 was found to inhibit biofilms of *P*. *aeruginosa* and MRPA CCARM 2095 under static and shaking conditions, respectively. Differences in the inhibitory effects of the peptide on bacterial cells versus biofilms were observed, with variations in experimental conditions among assays—including peptide contact and the cell/peptide ratio—potentially leading to differences in peptide activity. These differences are further illustrated by the changes in MIC and MBC values of *P*. *aeruginosa* under static and shaking conditions (Table S1). We employed µg/mL (micrograms per milliliter) concentration unit in the study as it is commonly used to convey the antimicrobial efficacy of conventional antibiotics and thereby offers practical applicability. Since it is important to take into account the exact bacterial cells, peptide concentrations, and volumes to further understand the antimicrobial functionality of PA-Win2, we provided factors from each experiment in Table S2.

In *P*. *aeruginosa*, three major QS systems—Las, Rhl, and PQS—regulate the expression of various genes involved in biofilm formation and bacterial pathogenicity [41]. At the top of this hierarchy, the *Las* system induces the production of molecules necessary for biofilm formation by activating the Rhl and PQS systems [42,43]. The Rhl system subsequently contributes to the structural stability of the biofilm by producing rhamnolipid and pyocyanin, while the PQS system is activated in the later stages of biofilm development, promoting cell dispersal and modulating toxin production [44]. Experimental results indicated that the peptide inhibits the biofilm formation of *P*. *aeruginosa* in a dose-dependent manner and effectively degrades established biofilms. Although a decreasing trend was observed, there was no noticeable inhibitory effect on the biofilm formation and suppression in MRPA. In terms of gene expression, PA-Win2 inhibited the QS genes of both *P*. *aeruginosa* and MRPA CCARM 2095. It can be speculated that biofilm formation and maturation in MRPA CCARM 2095 may occur through an alternative system, circumventing the effects of the peptide. The inhibition of biofilm and QS expression in *P*. *aeruginosa* by PA-Win2, along with its strong bactericidal effects, suggests that PA-Win2 can efficiently suppress the future emergence and spread of MRPA strains.

In this study, PA-Win2, exhibiting antibacterial and anti-biofilm activity, was successfully discovered using in silico methods, and its underlying mechanisms have been elucidated. While the research focused on the antimicrobial mechanisms of PA-Win2 against *P*. *aeruginosa* and MRPA CCARM 2095, further studies on a wider range of pathogenic bacteria are necessary. Additionally, to utilize peptides for therapeutic purposes, it is crucial to investigate the stability and biocompatibility of the peptide. In silico pipelines not only generate candidate peptide sequences from biological big data but also may promote optimization research, such as minimizing cytotoxicity, enhancing biological activity, and incorporating other desired functionalities. These AMPs may serve as an effective alternative and can be utilized for a combinatorial approach, exerting synergistic activities. In conclusion, PA-Win2 has been suggested as a potential therapeutic agent against bacterial infections, including those caused by *P*. *aeruginosa* and MRPA, where its discovery offers insights into the use of in silico pipelines for identifying useful resources from existing datasets as well as discovering biological value.

## 4. Materials and Methods

### 4.1. In Silico Methods Used for AMP Discovery

The venom gland transcriptome of *Pardosa astrigera* and the DL model from previous studies were utilized for the current research [22,23]. The 20-mer truncates produced along with prediction results of antimicrobial activities have been updated in https://github.com/bzlee-bio/AMPSpeciesSpecific (accessed on 11 November 2024). Peptide property calculator was used to calculate the net charge and water solubility of the sequences (https://pepcalc.com/, accessed on 2 October 2024). PEP-FOLD4 was used for the three-dimensional structural modeling of PA-Win2 (https://bioserv.rpbs.univ-paris-diderot.fr/services/PEP-FOLD4/, accessed on 4 October 2024) [45]. Helical features and AA arrangement were analyzed via the HELIQUEST program (https://heliquest.ipmc.cnrs.fr/cgi-bin/ComputParams.py/, accessed on 2 October 2024).

### 4.2. Peptide Synthesis and Preparation

Following the selection process, peptide PA-Win2 was synthesized by Biostem (Ansan, Republic of Korea). Peptide purity was obtained at >95% and was verified with high-performance liquid chromatography and mass spectroscopy (Figure S4). The peptides were resuspended in distilled water and peptide aliquots were stored at −80 °C before further use.

### 4.3. Peptide Stability Test

An equal amount of peptide was mixed in TSB and was incubated for 5 min, 10 min, 30 min, 1 h, 6 h, 12 h, and 24 h at 37 °C. The samples were separated by 10% SDS-polyacrylamide gel electrophoresis. Coomassie blue staining was performed for 30 min to visualize the peptide content in the gels. Gel was imaged after the destaining process for 2 h under shaking conditions.

### 4.4. Bacterial Strains and Cell Lines

Bacterial strains of *B*. *subtilis* ATCC 6051, *E*. *coli* KCCM 11234, *P*. *aeruginosa* ATCC 9027, *S*. *aureus* KCCM 11335, *S*. *epidermidis* ATCC 12228, and MRPA CCARM 2095 were used in this study. Each strain was grown overnight in TSB (Difco Laboratories, Detroit, MI, USA) at 37 °C under shaking conditions. hADMSCs were purchased from CEFO Co., (Seoul, Republic of Korea) and cultured in CEFOgro Human MSC Growth Medium (CEFO Co.) supplemented with 10% fetal bovine serum (FBS, Gibco, Grand Island, NY, USA), 1% penicillin/streptomycin (Gibco), and sodium pyruvate (Welgene, Gyeongsan, Republic of Korea). HaCaT (CLS 300493) and HDFα (ATCC PCS-201-012) cell lines were cultured in high glucose DMEM (Welgene) containing 10% FBS (Gibco) and 1% penicillin/streptomycin (Welgene). All cell lines were cultured in a humid incubator at 37 °C with 5% CO_2_.

### 4.5. Antimicrobial Activity Assays

MIC and MBC tests were conducted to evaluate the antibacterial activities of the peptide PA-Win2. To determine the MIC, we performed two-fold broth microdilution assays. The bacterial cultures were diluted into 4 × 10^5^ CFU/mL, and 50 μL of the dilutions were transferred into a 96-well plate. The peptides were diluted in CCW, and an equal volume of peptide samples was added to each well, resulting in final concentrations of peptides ranging from 0.25 to 64 µg/mL. The plates were incubated at 37 °C for 16 h under shaking conditions, and the absorbance was measured at 600 nm using a microplate reader (Molecular Devices, Sunnyvale, CA, USA). The MIC values were decided as the lowest concentration of the peptide without any observable bacterial growth. For evaluating the MBC values of the peptides, bacterial suspension samples above MIC values were spread onto tryptic soy agar (TSA, Difco Laboratories, Detroit, MI, USA) plates and incubated at 37 °C for 16 h. The MBC values were defined as the lowest peptide concentration at which no bacterial growth was observed.

### 4.6. Cell Viability Assay

Cell viability assay was conducted to evaluate the effect of PA-Win2 on hADMSC, HaCaT, and HDFα cells using the Quanti-Max WST-8 Cell Viability Assay Solution (WST-8 Solution, Bio-max, Seoul, Republic of Korea). Cells were seeded in 96-well plates at a density of 1 × 10^4^ cells/well and treated with various concentrations of the peptide for 24 h. After incubation, the WST-8 solution was added to each well, and the plate was incubated at 37 °C for 30 min. Absorbance was measured at 450 nm using a microplate reader (Molecular Devices), and cell viability was presented relative to the control

### 4.7. Time-Kill Curve Assay

A time-kill curve assay was conducted to assess the effect of peptide treatment on bacterial growth over time. The bacterial culture was diluted into 4 × 10^5^ CFU/mL in TSB, and 1.5 mL of the suspension was transferred into 15 mL conical tubes. An equal volume of peptides was added, and the mixture was incubated at 37 °C with shaking. Samples were taken every hour for 6 h, diluted 1/400, and spread onto TSA plates. The plates were incubated overnight at 37 °C. Visible colonies on the agar plates were counted, and the relative colony formation was compared with that of the control plate.

### 4.8. Membrane Depolarization Measurement

To evaluate cytoplasmic membrane permeation by the peptide, a DiSC_3_(5) release assay was performed. Bacteria were cultured in TSB overnight at 37 °C and then diluted to 1 × 10⁷ CFU/mL. The samples were washed three times with 5 mM HEPES buffer and resuspended in the same buffer containing 0.4 μM DiSC_3_(5). A 100 μL aliquot of the bacterial suspension was transferred into each well of a black 96-well microplate and incubated in the dark for 30 min at 37 °C. Following incubation, an equal volume of peptides or 0.5% SDS (Biosolution, Seoul, Republic of Korea) as a positive control for 100% membrane disruption was added. Fluorescence was measured for 10 min using an Infinite F200 Pro multimode microplate reader (Tecan, Männedorf, Switzerland) with an excitation wavelength of 622 nm and an emission wavelength of 670 nm. The fluorescence intensity was presented by subtracting the baseline value from the HEPES buffer-treated group.

### 4.9. RT-qPCR

Total mRNA was isolated from *P*. *aeruginosa* and MRPA CCARM 2095 using TRizol Reagent (Invitrogen, Thermo Fisher Scientific, Waltham, MA, USA). After determining the concentration of mRNA using Nanodrop-2000 (Thermo Fisher Scientific), 2 μg of the extracted mRNA was used to synthesize using M-MLV Reverse transcriptase (ELPISBIO, Daejeon, Republic of Korea). The reaction mixture contained 7 μL of distilled water, 10 μL of SYBR Green PCR Master Mix, 1 μL of each forward and reverse primer, and 1 μL of synthesized cDNA. The primer used in this study is listed in Table S3. The RT-qPCR was performed under the following thermocycling conditions: pre-denaturation at 95 °C for 3 min, denaturation at 95 °C for 10 s, annealing at 60 °C for 30 s, and extension at 72 °C for 30 s. The reaction was conducted using the CFX Connect Real-Time PCR Detection System (Bio-Rad, Hercules, CA, USA) and was repeated for 40 cycles.

### 4.10. Biofilm Formation and Inhibition Assay

CV staining assays were conducted to evaluate the inhibitory effect of the peptide on biofilm formation or inhibition. For the biofilm formation assay, a 22 × 22 mm glass coverslip was placed in each well of 6-well plates, then sterilized with 70% ethanol and UV exposure. Bacteria were seeded at a density of 2 × 10⁵ CFU/well, and 100 μL of peptides were added to each well at final concentrations of 1, 2, 4, or 8 μg/mL. After 24 h of incubation, the biofilms were carefully washed with phosphate-buffered saline (PBS, Biosolution) and stained with 1 mL of CV working solution (0.25% CV in 20% ethanol). Following a 15 min incubation at room temperature, samples were washed twice with PBS. Coverslips were transferred to new 6-well plates and air-dried. After imaging, the stained dye was eluted with 100% methanol (Sigma-Aldrich, Saint Louis, MO, USA). A 100 μL aliquot of the eluted samples was transferred to a 96-well plate, and absorbance was measured at 570 nm using a microplate reader (Molecular Devices) for quantitative analysis.

For the biofilm inhibition assay, bacteria samples of 2 × 10⁵ CFU/well were seeded onto sterilized glass coverslips and cultured for 3 d at 37 °C. Subsequently, 100 μL of peptides was added to each well, and the plates were incubated for an additional 3 h. CV staining was performed as described above, with biofilms imaged and quantified.

### 4.11. Statistical Analysis

All experiments were conducted at least three times to produce triplicates from cases. The data distribution was tested using the Shapiro–Wilk test prior to analyses. The results are expressed as the mean ± standard error of the mean (SEM). The statistical analyses were performed using either a *t*-test or a one-way ANOVA test followed by Tukey’s post-test. Non-parametric data testing was performed using the Mann–Whitney test and the Kruskal–Wallis tests, followed by Dunn’s post hoc test. All statistical evaluations were conducted using GraphPad Prism 10.0.0 (GraphPad Software, La Jolla, CA, USA). A *p*-value less than 0.05 was considered to indicate a statistically significant difference.

## Figures and Tables

**Table 1:** The five sequences with the highest antimicrobial functionality prediction scores.

Transcript ID	20-Mer Sequence	Antibacterial Activity Prediction (%)					Net Charge	Water Solubility
		*Bacillus subtilis*	*Escherichia coli*	*Pseudomonas aeruginosa*	*Staphylococcus aureus*	*Staphylococcus epidermidis*		
TBIU038741	IILLIIIILVVIYYRRRLRR	0.994	0.990	0.991	0.995	0.981	+5	Poor
TBIU034561	LILFRFLGYIVLRYVRKPK	0.994	0.989	0.990	0.995	0.979	+5	Poor
TBIU038389	LFLLIFCLWKLGFFKRRKPG	0.994	0.988	0.990	0.995	0.979	+4.9	Poor
TBIU038647	LLRGLRYLCLKILYILKLRK	0.994	0.988	0.990	0.994	0.979	+5.9	Good
TBIU038959	LILLIIILWKCGFFKRKKPG	0.994	0.987	0.990	0.994	0.979	+4.9	Poor

**Table 2:** MIC and MBC values against tested bacterial strains.

Concentration (μg/mL)	*Bacillus subtilis* ATCC 6051		*Escherichia coli* KCCM 11234		*Pseudomonas* *aeruginosa* ATCC 9027		*Staphylococcus aureus* KCCM 11335		*Staphylococcus epidermidis* ATCC 12228		MRPA CCARM 2095	
	MIC	MBC	MIC	MBC	MIC	MBC	MIC	MBC	MIC	MBC	MIC	MBC
Ampicillin	8	8	64	128	256	>256	0.125	1	32	64	>512	>512
Streptomycin	128	>256	8	16	32	32	8	16	>512	>512	>512	>512
Tetracycline	2	32	0.5	32	32	>256	0.125	>32	8	64	>512	>512
Rifampicin	1	8	8	16	16	>16	0.125	0.5	0.125	0.125	16	16
PA-Win2	2	2	8	32	4	4	256	>256	64	>256	2	2

## Data Availability

The original contributions presented in the study are included in the article/Supplementary Material, further inquiries can be directed to the corresponding author.

## Supplementary Materials

The following supporting information can be downloaded at: https://www.mdpi.com/article/10.3390/antibiotics13121113/s1, Figure S1: Peptide stability in tryptic soy broth (TSB), Figure S2: Analysis of biofilm formation and inhibition effect of PA-Win2 on MRPA CCARM 2095 under static condition, Figure S3: Selection of bacterial concentrations for biofilm formation under shaking condition, Figure S4: Quality control of synthesized PA-Win2 by high-performance liquid chromatography (HPLC) and mass spectroscopy (MS), Table S1: MIC and MBC values of *Pseudomonas aeruginosa* ATCC 9027 according to the incubation conditions, Table S2: The number of bacterial cells, peptide concentrations, and volume of each experiment, Table S3: Primer sequences used in the study.
