# Supplementary material for: PA-Win2: In Silico-Based Discovery of a Novel Peptide with Dual Antibacterial and Anti-Biofilm Activity
Source: Antibiotics (Basel). 2024 Nov 21;13(12):1113. doi: 10.3390/antibiotics13121113 (PMC11672609; doi:10.3390/antibiotics13121113)
Supplement: Supplementary file 1 [file antibiotics-13-01113-s001.zip › antibiotics-3288224-supplementary.pdf]

Supplementary Materials

PA-Win2: In silico-based discovery of a novel peptide with dual antibacterial and anti-biofilm activity

Jin Wook Oh [1,†], Min Kyoung Shin [1,†], Hye-Ran Park [1], Sejun Kim [1], Byungjo Lee [2], Jung Sun Yoo [3], Won-Ja Chi [4] and Jung-Suk Sung [1,*]

1 Department of Life Science, Dongguk University-Seoul, Goyang 10326, Republic of Korea; oh5929@dongguk.edu (J.W.O.); shinmk94@dgu.ac.kr (M.K.S.); 2019111678@dongguk.edu (H.-R.P.); tpwns413@dongguk.edu (S.J.)
2 Research Institute, National Cancer Center, Goyang, 10408, Republic of Korea; blee.inf@gmail.com
3 Wildlife Quarantine Center, National Institute of Wildlife Disease Control and Prevention, Incheon 22382, Republic of Korea; lycosidae@korea.kr
4 Species Diversity Research Division, National Institute of Biological Resources, Incheon 22689, Republic of Korea; wjchi76@korea.kr
* Correspondence: sungjs@dongguk.edu; Tel.: +82-31-961-5132
† These authors contributed equally to this work.

Figure S1. Peptide stability in tryptic soy broth (TSB).

Figure S2. Analysis of biofilm formation and inhibition effect of PA-Win2 on MRPA CCARM 2095 under static condition.

Figure S3. Selection of bacterial concentrations for biofilm formation under shaking condition.

Figure S4. Quality control of synthesized PA-Win2 by high-performance liquid chromatography (HPLC) and mass spectroscopy (MS).

Table S1. MIC and MBC values of Pseudomonas aeruginosa ATCC 9027 according to the incubation conditions.

Table S2. The number of bacterial cells, peptide concentrations, and volume of each experiment.

Table S3. Primer sequences used in the study.

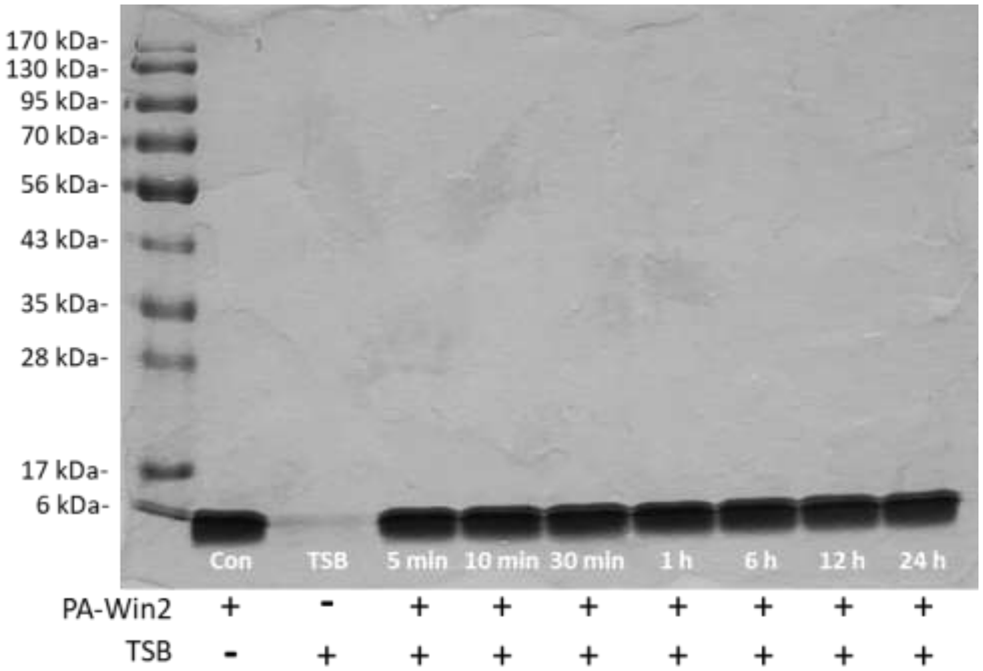

Figure S1. Peptide stability in tryptic soy broth (TSB).
To assess the stability of PA-Win2 in TSB, sodium dodecyl sulfate-polyacrylamide gel electrophoresis and Coomassie blue staining were performed. The peptides were incubated in TSB for 5 min, 10 min, 30 min, 1 h, 6 h, 12 h, and 24 h. The samples were separated by SDS-PAGE, and the gel was stained with Coomassie blue dye and imaged after destaining process.

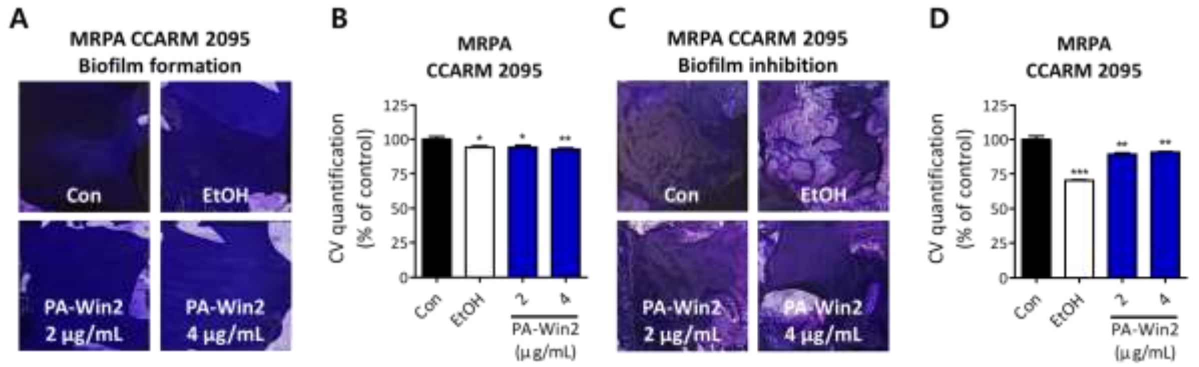

Figure S2. Analysis of biofilm formation and inhibition effect of PA-Win2 on MRPA CCARM 2095 under static conditions.

The biofilms were stained by crystal violet (CV), and representative images of (A) biofilm formation and (C) inhibition in MRPA CCARM 2095 were presented. The stained biofilms were eluted using methanol and quantified relative to the control. (B, D) The data for biofilm formation and inhibition were illustrated by bar graphs. * $p < 0.05$, ** $p < 0.01$ and *** $p < 0.001$ compared with control group. Con: Control, EtOH: Ethanol.

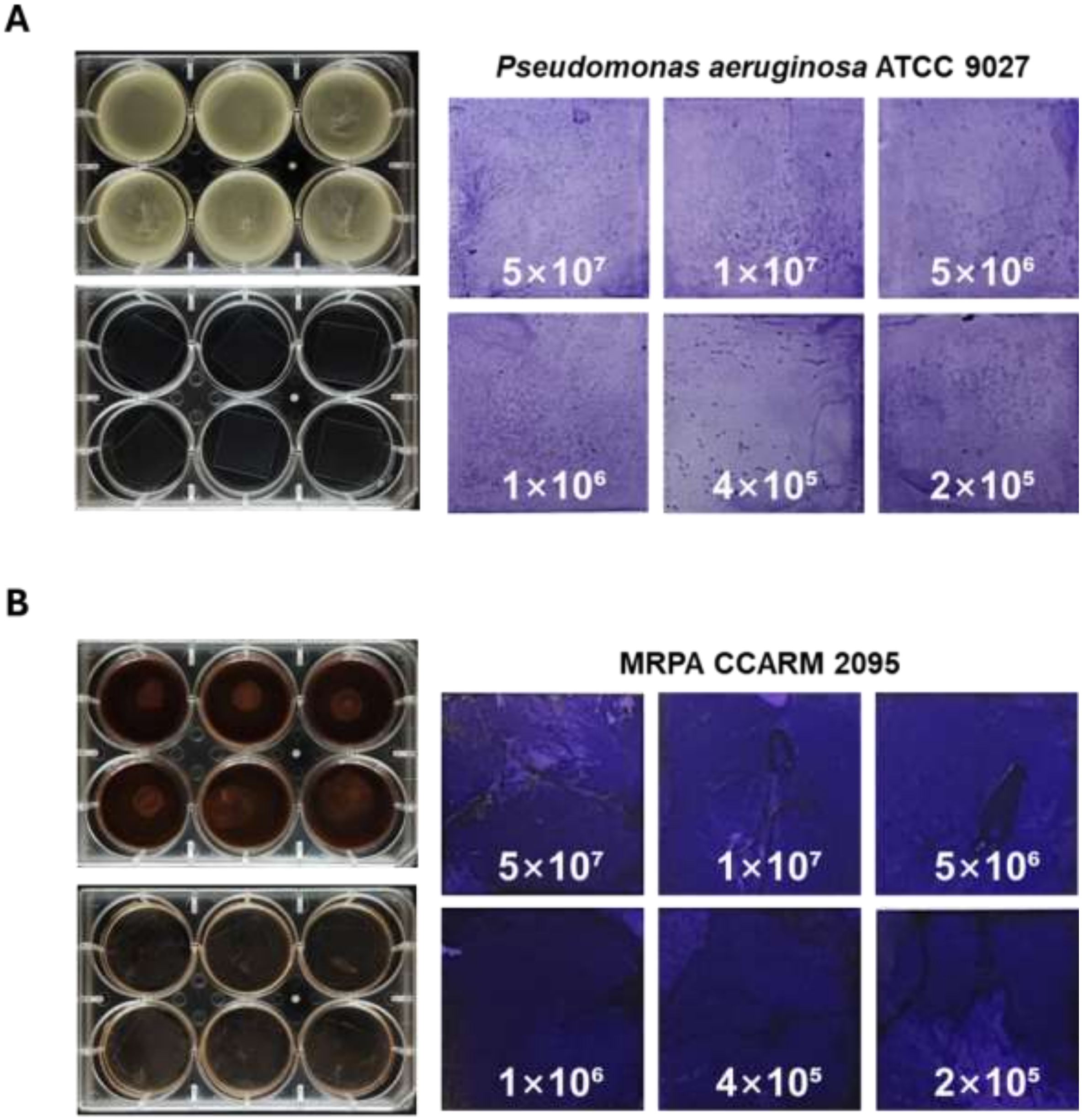

Figure S3. Selection of bacterial concentrations for biofilm formation under shaking condition. Various bacterial concentrations were tested to establish biofilm formation conditions of *Pseudomonas aeruginosa* ATCC 9027 and multi-drug resistant *Pseudomonas aeruginosa* (MRPA) CCARM 2095 under shaking condition. Bacterial cultures were inoculated at 5×10^7, 1×10^7, 5×10^6, 1×10^6, 4×10^5, and 2×10^5 CFU/well and incubated for 24 h with shaking. Results indicated that (A) MRPA CCARM 2095 formed biofilms across all bacterial concentrations, whereas (B) *P. aeruginosa* ATCC 9027 did not form biofilms at every concentration. Based on these observations, a concentration of 2×10^5 CFU/well was selected as the optimal biofilm formation condition for MRPA CCARM 2095 under shaking conditions.

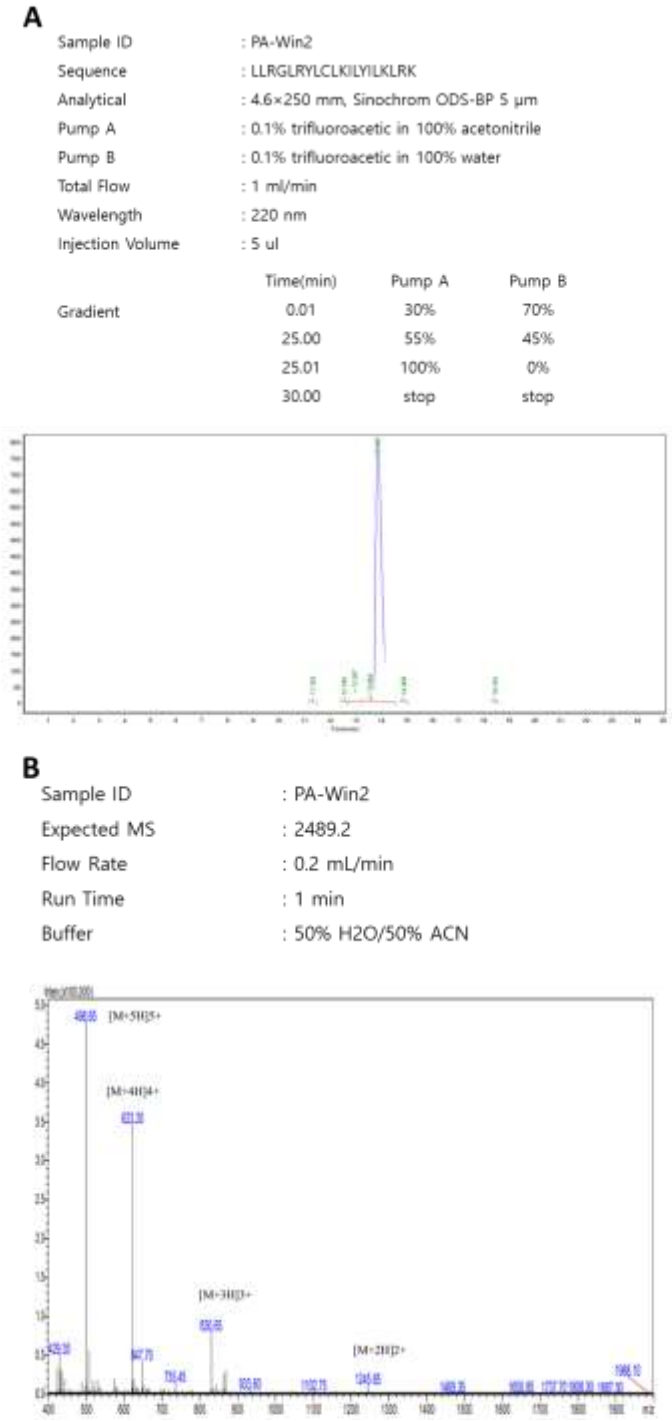

Figure S4. Quality control of synthesized PA-Win2 by high-performance liquid chromatography (HPLC) and mass spectroscopy (MS).

(A) HPLC profile and (B) MS chromatogram of PA-Win2 were shown. The peptide was collected with a purity >95%.

Table S1. MIC and MBC values of *Pseudomonas aeruginosa* ATCC 9027 according to the incubation conditions.

μg/mL	*Pseudomonas aeruginosa* ATCC 9027	
	MIC	MBC
Shaking condition	4	4
Static condition	4	8

Table S2. The number of bacterial cells, peptide concentrations, and volume of each experiment.

Assay	Bacterial cell number	Bacterial strain	Peptide concentration (dilution ratio: 20)		Total volume
			μg/mL	μM	
MIC test	2 × 10^4 CFU	*B. subtilis* ATCC 6051	2	0.08	100 μL
		E. coli KCCM 11234	8	0.32	
		P. aeruginosa ATCC 9027	4	0.16	
		MRPA CCARM 2095	2	0.08	
Time-kill curve assay	6 × 10^5 CFU	*B. subtilis* ATCC 6051	2	2.4	3 mL
		E. coli KCCM 11234	8	9.6	
		P. aeruginosa ATCC 9027	4	4.8	
		MRPA CCARM 2095	2	2.4	
$DiSC_3(5)$ release assay	2 × 10^7 CFU	*B. subtilis* ATCC 6051	1	0.08	200 μL
			2	0.16	
		E. Coli KCCM 11234	4	0.32	
			8	0.64	
		P. aeruginosa ATCC 9027	2	0.16	
			4	0.32	
		MRPA CCARM 2095	1	0.08	
			2	0.16	
RT-qPCR	2 × 10^8 CFU	*P. aeruginosa* ATCC 9027	4	3.2	2 mL
		MRPA CCARM 2095	2	1.6	
Biofilm formation RT-qPCR (static condition)	2 × 10^8 CFU	*P. aeruginosa* ATCC 9027	4	3.2	2 mL

**Biofilm inhibition RT-qPCR (static condition)**	**2 × 10^5 CFU**	***P. aeruginosa* ATCC 9027**	**4**	**3.2**	**2 mL**
**Biofilm formation RT-qPCR (shaking condition)**	**2 × 10^8 CFU**	**MRPA CCARM 2095**	**1**	**0.8**	**2 mL**
**Biofilm inhibition RT-qPCR (shaking condition)**	**2 × 10^5 CFU**	**MRPA CCARM 2095**	**1**	**0.8**	**2 mL**
**Biofilm formation assay (static condition)**	**2 × 10^5 CFU**	***P. aeruginosa* ATCC 9027**	**4**	**3.2**	**2 mL**
			**8**	**6.4**	
		**MRPA CCARM 2095**	**2**	**1.6**	
			**4**	**3.2**	
**Biofilm inhibition assay (static condition)**	**2 × 10^5 CFU**	***P. aeruginosa* ATCC 9027**	**4**	**3.2**	**2 mL**
			**8**	**6.4**	
		**MRPA CCARM 2095**	**2**	**1.6**	
			**4**	**3.2**	
**Biofilm formation assay (shaking condition)**	**2 × 10^5 CFU**	**MRPA CCARM 2095**	**1**	**0.8**	**2 mL**
			**2**	**1.6**	
**Biofilm inhibition assay (shaking condition)**	**W2 × 10^5 CFU**	**MRPA CCARM 2095**	**1**	**0.8**	**2 mL**
			**2**	**1.6**	

Table S3. Primer sequences used in the study.

Gene	Forward primer sequence (5′ → 3′)	Reverse primer Sequence (5′ → 3′)
16s rRNA	CAAAACTACTGAGCTAGAGTACG	TAAGATCTCAAGGATCCCAACGGCT
gryA	GTGTGCTTTATGCCATGAG	GGTTTCCTTTTCCAGGTC
MurD	GTAGCGATCCATATGGTCTTCG	ACATCGAGCTGTACGTGTTG
parC	CATCGTCTACGCCATGAG	AGCAGCACCTCGGAATAG
Pbp2	GCCCAACTACGACCACAAG	CGCGAGGTCGTAGAAATAG
polA	TCAACACCATGACCGGTAGC	GGGGATGTTGTCGACCTTGT
rpoB	CTGATCATCTTCGACCGCGA	TTCTCGGTCATCAGGGGGAT
rpsL	GCTGCAAAACTGCCCGCAACG	ACCGCAGGTGTCCAGCGAACC
LasI	CGCACATCTGGGAACTCA	CGGCACGGATCATCATCT
LasR	GCAGCACGAGTTCTTCGAGG	GCGTAGTCCTTGAGCATCCAC
PqsA	GACCGGCTGTATTCGATTC	GCTGAACCAGGGAAAGAAC
PqsR	CTGATCTGCCGGTAATTGG	ATCGACGAGGAACTGAAGA
RhlI	TTCATCCTCCTTTAGTCTTCCC	TTCCAGCGATTCAGAGAGC
RhlR	TGCATTTTATCGATCAGGGC	CACTTCCTTTTCCAGGACG
